# Usp14 Deficiency Increases Tau Phosphorylation without Altering Tau Degradation or Causing Tau-Dependent Deficits

**DOI:** 10.1371/journal.pone.0047884

**Published:** 2012-10-29

**Authors:** Youngnam N. Jin, Ping-Chung Chen, Jennifer A. Watson, Brandon J. Walters, Scott E. Phillips, Karen Green, Robert Schmidt, Julie A. Wilson, Gail V. Johnson, Erik D. Roberson, Lynn E. Dobrunz, Scott M. Wilson

**Affiliations:** 1 Department of Neurobiology, Civitan International Research Center, University of Alabama at Birmingham, Birmingham, Alabama, United States of America; 2 Division of Neuropathology, Department of Pathology and Immunology, Washington University School of Medicine, Saint Louis, Missouri, United States of America; 3 Department of Anesthesiology, University of Rochester, Rochester, New York, United States of America; 4 Department of Neurology, University of Alabama at Birmingham, Birmingham, Alabama, United States of America; Brigham and Women's Hospital, Harvard Medical School, United States of America

## Abstract

Regulated protein degradation by the proteasome plays an essential role in the enhancement and suppression of signaling pathways in the nervous system. Proteasome-associated factors are pivotal in ensuring appropriate protein degradation, and we have previously demonstrated that alterations in one of these factors, the proteasomal deubiquitinating enzyme ubiquitin-specific protease 14 (Usp14), can lead to proteasome dysfunction and neurological disease. Recent studies in cell culture have shown that Usp14 can also stabilize the expression of over-expressed, disease-associated proteins such as tau and ataxin-3. Using Usp14-deficient *ax^J^* mice, we investigated if loss of Usp14 results in decreased levels of endogenous tau and ataxin-3 in the nervous system of mice. Although loss of Usp14 did not alter the overall neuronal levels of tau and ataxin-3, we found increased levels of phosphorylated tau that correlated with the onset of axonal varicosities in the Usp14-deficient mice. These changes in tau phosphorylation were accompanied by increased levels of activated phospho-Akt, phosphorylated MAPKs, and inactivated phospho-GSK3β. However, genetic ablation of tau did not alter any of the neurological deficits in the Usp14-deficient mice, demonstrating that increased levels of phosphorylated tau do not necessarily lead to neurological disease. Due to the widespread activation of intracellular signaling pathways induced by the loss of Usp14, a better understanding of the cellular pathways regulated by the proteasome is required before effective proteasomal-based therapies can be used to treat chronic neurological diseases.

## Introduction

The ubiquitin proteasome system (UPS) functions to control intracellular protein abundance [1] and is tightly regulated to facilitate the removal of damaged proteins that can cause disease [2]–[4]. Cellular proteins are marked for degradation by tagging them with a 76 amino acid protein called ubiquitin [5], enabling them to productively interact with the proteasome, a multi-subunit protease which catalyzes their breakdown into small peptides. Changes in the targeting and destruction of ubiquitinated proteins are observed in many chronic neurological diseases, reinforcing the importance of regulated proteolysis in neuronal viability and function [6], [7].

Following the binding of ubiquitinated substrates to the proteasome, ubiquitin side chains can be disassembled and/or modified by further ubiquitination [8]. The proteasomal factors responsible for these activities are believed to either enhance or antagonize substrate degradation. For example, by removing ubiquitin side chains prior to commitment of substrates to degradation, proteasomal deubiquitinating enzymes can prevent substrates from being degraded by the proteasome [9], [10]. Usp14 is one of the proteasomal deubiquitinating enzymes that can remove ubiquitin side chains on proteins bound to the proteasome and, as such, plays an important role in ubiquitin recycling and substrate stability [11].

Our previous studies demonstrated that the loss of Usp14 expression specifically in the nervous system causes *ax^J^* mice to display a resting tremor, gait ataxia, motor-endplate disease and cerebellar pathology due to impaired neuronal development [12], [13]. It is currently unknown if Usp14 is dispensable in the adult nervous system. Although the *ax^J^* mice have dysfunctional proteasomes, there is no accumulation of ubiquitinated proteins or increase in apoptotic cells in the central nervous system [13]. This observation is consistent with the finding that loss of Usp14 results in an increase in the turnover of ubiquitinated proteins *in vitro* [14], [15]. Loss of Usp14 in cell culture models resulted in a decrease in the steady-state levels of aggregate-prone proteins such as tau and ataxin-3 [14], indicating that Usp14 may serve as a therapeutic target for the treatment of neurodegenerative proteinopathies [14].

The association of elevated ubiquitin conjugates with intracellular deposits of aggregate-prone proteins suggests a causative link between proteasome dysfunction and disease [16]. For example, tau accumulation can lead to the production of abnormally modified oligomeric tau and neurofibrillary tangles (NFTs) that may contribute to neurological disease in patients with Alzheimer's disease (AD) [17]. Since reduced proteasomal activity and increased tau accumulation has been observed in the brains of AD patients [18], enhancing the activity of the proteasome may offer a potential therapeutic avenue for the treatment of diseases associated with the accumulation of aggregate-prone proteins.

Proteasome inhibition has been shown to alter the expression of several mitogen-activated signaling pathways [19], [20]. By selectively targeting protein kinases for degradation, the UPS is able to directly control intracellular signaling events. For example, ASK-1, the mitogen-activated protein kinase kinase kinase, is ubiquitinated and targeted to the proteasome by the E3 ligase CHIP [21]. In addition, proteasome inhibition has also been shown to induce c-Jun NH_2_-terminal kinase (JNK) and extracellular signal-regulated kinases (ERKs) [19], [22]. While proteasome inhibition has been shown to have both pro- and anti-apoptotic effects, the result of enhanced proteasome function on intracellular signaling pathways is currently unknown [22], [23].

In this study, we investigated if loss of Usp14 *in vivo* affects the levels of aggregate-prone proteins in the central nervous system. Although loss of Usp14 did not alter the steady-state levels of either endogenous tau or ataxin-3 in mice, we did observe a significant increase in the levels of phosphorylated tau and increased activation of Akt, JNK and ERK. Genetic ablation of tau did not change the disease course in the *ax^J^* mice, indicating that abnormal tau phosphorylation was not responsible for causing the neurological deficits in the *ax^J^* mice. Our results therefore suggest that the expression of the aggregate-prone proteins tau and ataxin-3 are not controlled by Usp14 in neurons and that the loss of Usp14 activates several different neuronal signaling pathways that may contribute to the synaptic transmission defects observed in the *ax^J^* mice.

## Methods

### Ethics Statement

The experiments described in this manuscript were approved by the University of Alabama at Birmingham Institutional Animal Care and Use Committee (IACUC). All research complied with the United States Animal Welfare Act and other Federal statutes and regulations relating to animals and experiments involving animals, and adhered to principles stated in the *Guide for the Care and Use of Laboratory Animals*, United States National Research Council. All reasonable efforts were made to minimize suffering of animals.

### Animals

Wild type (wt) C57BL/6J and Usp14*^axJ^* mice (Jackson Laboratories, Bar Harbor, ME) have been maintained in our breeding colony at the University of Alabama at Birmingham, which is fully accredited by the Association for Assessment and Accreditation of Laboratory Animal Care International (Animal Protocol number 110909471). Mice were housed with a 12 hr light/dark cycle in ventilated cages and maintained on Harlan Teklad 7904 breeders' diet. Homozygous Usp14*^axJ^* mice, which we refer to as *ax^J^* mice, were generated by intercrossing *ax* ^J^/+ siblings. Tau deficient mice (*tau^KO^*) were obtained from Jackson Laboratories [24]. The *ax^J^* mice were crossed to *tau^KO^* mice to generate mice homozygous for the *ax^J^* and *tau* mutations (*ax^J^tau^KO^*). Mice were euthanized by CO_2_ asphyxiation.

### Body mass analysis

Body weights were collected from 4 and 8-week-old *wt*, *ax^J^*, *tau^KO^* and *ax^J^tau^KO^* mice. Weights were determined for 4 animals per genotype. Values represent the average body mass ± SE.

### Electron Microscopy

Animals were anesthetized with ketamine/xylazine and perfused with 25 mL of heparinized saline followed by 25 mL of modified Karnovsky's fixative containing 3% glutaraldehyde and 1% paraformaldehyde in sodium cacodylate buffer, pH 7.4. Fixation was continued overnight at 4°C in the same fixative, and the following day the cerebellum was dissected, cleaned of extraneous tissue, and rinsed in sodium cacodylate buffer. Tissue was post-fixed in phosphate cacodylate-buffered 21% OsO_4_ for 1 h, dehydrated in graded ethanol with a final dehydration in propyleneoxide, and embedded in EMbed-812 (Electron Microscopy Sciences, Hatfield, PA). One-micron-thick plastic sections were examined by light microscopy after staining with toluidine blue. Ultrathin sections (90 nm thick) were cut onto formvar-coated slot grids. Sections were post-stained with uranyl acetate and Venable's lead citrate and viewed with a 1200EX electron microscope (JEOL, Tokyo, Japan). Digital images were acquired using the AMT Advantage HR camera (Advanced Microscopy Techniques, Danvers, MA).

### Quantitation of Purkinje cell axonal swellings

Four to five 5 to 6-week-old wt, *ax^J^*, *tau^KO^* and *ax^J^tau^KO^* mice were euthanized by CO_2_ asphyxiation. Brains were sagittally cut and were immersed in methacarne to fix overnight at 4°C. Samples were paraffin embedded and sliced medially in 10 µm sections with a Microme HM 355S. Twenty medial sections from each brain were taken, and three sections from each sample were randomly chosen to quantitate. Chosen sections were deparaffinized and rehydrated. Sections were blocked in a 10 mM PBS solution containing 1% BSA, 0.2% non-fat dry milk, 0.1% Triton X-100 for 30 min, and were then incubated with a 1∶500 dilution of a rabbit calbindin polyclonal antibody (Swant, Bellinzona, Switzerland) for 1 h at room temperature. Samples were washed 3 times with 10 mM PBS for 5 min. The sections were then treated with Alexa-Fluor goat-anti-rabbit 568 at a 1∶500 dilution and DAPI at a 1∶1000 dilution in the dark for 1 h at room temperature. Sections were washed 3 times with 10 mM PBS for 5 min. Samples were mounted with 50% glycerol in 10 mM PBS and stored at −20°C. An Olympus BX-51 upright microscope was utilized to take images at 10× of the medial deep cerebellar nuclei. Axonal swellings greater than 2 mm were counted and normalized to the area of the cerebellar nuclei region with the MicroBrightField Stereo Investigator software (Williston, VT).

### Electrophysiology

Hippocampal slices were held in a submersion recording chamber perfused with external recording solution composed of (in mM): 120 NaCl, 3.5 KCl, 2.5 CaCl_2_, 1.3 MgCl_2_, 1.25 NaH_2_PO_4_, 26 NaHCO_3_, and 10 glucose [25]. The solution was bubbled with 95% O_2_ and 5% CO_2_, pH 7.35–7.45. All experiments were performed at 25°C.

Field excitatory postsynaptic potentials (fEPSPs) were recorded in response to extracellular stimulation of Schaffer collateral axons by a bipolar tungsten microelectrode (FHC, Bowdoinham, ME) placed in the stratum radiatum of the CA1 region. Recording pipettes filled with external recording solution were placed in the same region of the CA1 stratum radiatum. Stimulation was generated from a Grass S48 stimulator (Grass Tech, West Warwick, RI) and applied with a BSI-2 biphasic stimulus isolator (BAK Electronics, Mount Airy, MD).

Paired-pulse stimulation at different intervals (50, 100, 150, 200, and 500 ms) was applied in a pseudo-random sequence and repeated a minimum of 10 times at 0.1 Hz. The averaged paired-pulse ratio (fEPSP_2_/fEPSP_1_) was calculated after recording.

### PHF-1 Staining

Paraffin-embedded sections of mouse brains (7 µm) were deparaffinized and followed with antigen retrieval by citrate buffer (10 mM, pH 6). The endogenous peroxidase activity was blocked with 3% hydrogen peroxide. Sections were blocked with 3% goat serum for 30 min and were incubated with the PHF-1 antibody (1∶500) overnight at 4°C. The PHF-1 antibody recognizes tau phosphorylated at Ser 396/404. Sections were then incubated with biotinylated goat secondary antibody for 30 min, and then incubated with avidin-biotin peroxidase complex (Vector Laboratories, Burlingame, CA) for 60 min at room temperature. Diaminobenzidine (DAB) was used for color development and was followed with hematoxylin counterstain.

### Immunoblotting

Proteins were resolved on 8% Tris-glycine gels and transferred onto PVDF membranes. The Usp14 R138 antisera [12], ataxin-3 (gift from Dr. Hank Paulson, University of Michigan), PHF-1 antibody (gift from Dr. Peter Davis, Albert Einstein College of Medicine), tau1 (dephosphorylated amino acids 189–207), tau5 (amino acids 210–230), 12E8 (pSer262/pSer356), AT180 (pThr231), AT100 (pThr212/pSer214) (gifts from Dr. Gail Johnson, Rochester University), anti-β-tubulin, and anti-α-tubulin (Developmental Hybridoma Core, Iowa City, IA), GSK3β, Akt, Cdk5, p35, MEK1/2, JNK1/2 and ERK1/2 (Cell Signaling, Danvers, MA) antibodies were diluted in PBS containing 5% non-fat milk. Primary antibodies were detected using an anti-mouse or anti-rabbit HRP-conjugated antibody (Southern Biotechnology Associates, Birmingham, AL) and Luminol reagents (Pierce, Rockford, IL).

### Quantitation of immunoblots

Blots were scanned using a Hewlett Packard Scanjet 3970 and quantitated using UN-SCAN-IT software (Orem, UT). Each value represents the average and standard error from 4 blots using at least three different animals of each genotype.

## Results

### Tau and ataxin-3 expression in Usp14-deficient mice

**Figure 1:**
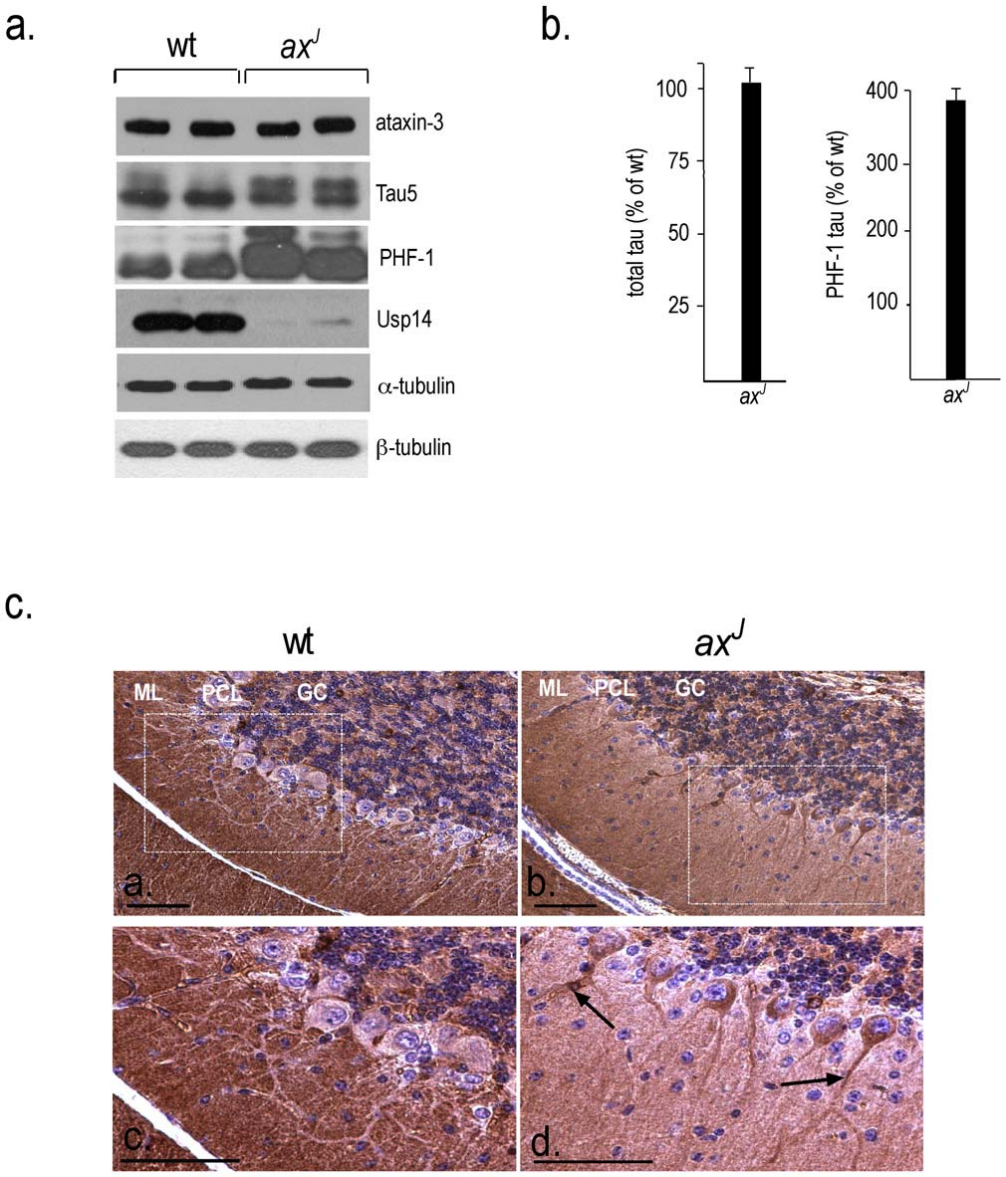
Steady-state levels of aggregate-prone tau and ataxin-3 proteins in the cerebellum of wt and Usp14-deficient *ax^J^* mice. (A) Representative immunoblot of ataxin-3, tau, α- and β-tubulin, and Usp14 from the cerebellum of two different 4-week-old wt and *ax^J^* mice. The tau5 antibody was used to determine total tau levels, and the PHF-1 antibody was used to identify phosphorylated tau. (B) Quantitation of total and PHF-1-reactive tau. n = 4 to 5 animals per genotype (C) Immunohistochemistry of PHF-1-reactive tau in the cerebellum of wt and *ax^J^* mice. Cryosections from each genotype were stained with the PHF-1 antibody and visualized using DAB. The boxed area in a and b is enlarged in c and d, respectively. Arrows indicate localized regions of intense staining with PHF-1 in Purkinje cell dendrites. ML, molecular layer; PCL, Purkinje cell layer; and GC, granule cell layer. Scale bars 50 µm.

We have recently reported that Usp14 is required for the stable expression of neurological disease-related proteins, such as tau and ataxin-3, in mouse embryonic fibroblasts [14]. To determine if Usp14 also stabilizes these proteins *in vivo*, we used immunoblot analysis to analyze the expression of endogenous tau and ataxin-3 in wt and *ax^J^* mice. In contrast to what was observed *in vitro* [14], we found no differences in the levels of ataxin-3 in cerebellar extracts from 4-week-old wt and *ax^J^* mice (Fig. 1a), indicating that Usp14 was not required for the neuronal stability of ataxin-3. Although immunoblot analysis demonstrated that the overall levels of tau were also similar in the wt and *ax^J^* mice (Fig. 1a and b), the absence of Usp14 did result in a shift in the migration pattern of tau in the *ax^J^* mice (Fig. 1a). To determine if the slower migrating form of tau found in the cerebellar extracts from the *ax^J^* mice represents increased levels of phospho-tau, we performed immunoblot analysis on cerebellar extracts from wt and *ax^J^* mice using the phospho-specific tau antibody PHF-1 [26]. There was a significant increase in the expression of PHF-1-reactive tau in the *ax^J^* mice as compared to controls (Fig. 1a and 1b), demonstrating that, while loss of Usp14 does not affect the steady-state levels of tau in neurons, loss of Usp14 alters the phosphorylation state of tau in the *ax^J^* mice.

Slower migrating forms of tau have also been detected in the brains of AD patients, and these forms of tau are believed to be due to hyperphosphorylated tau, which may aggregate to form neurofibrillary tangles [17], [27]. Mislocalization of tau to the somatodendritic compartment of neurons instead of the axon is one of the earliest aspects of tau pathology [28]. To determine if the increased levels of phospho-tau observed in the *ax^J^* mice were associated with aberrant localization of tau, we stained cerebellar sections from wt and *ax^J^* mice with the PHF-1 antibody (Fig. 1c). The cerebellar sections from control mice revealed widespread PHF-1 staining in the molecular and granule cell layers (Fig. 1c). In contrast, the *ax^J^* mice exhibited strong PHF-1 staining in the Purkinje cell dendrites, cell body, and axonal swellings near the deep cerebellar nuclei and showed reduced staining in the molecular layer. Focal staining with PHF-1 was also noted in the dendrites of the *ax^J^* Purkinje cells (Fig. 1c). Increased PHF-1 staining was also detected in the cortex and hippocampus of the *ax^J^* mice (data not shown). Therefore, in addition to increasing the levels of phosphorylated tau in the neurons of the *ax^J^* mice, loss of Usp14 also leads to a redistribution of PHF-1-reactive tau in the Purkinje cells of the *ax^J^* mice.

**Figure 2:**
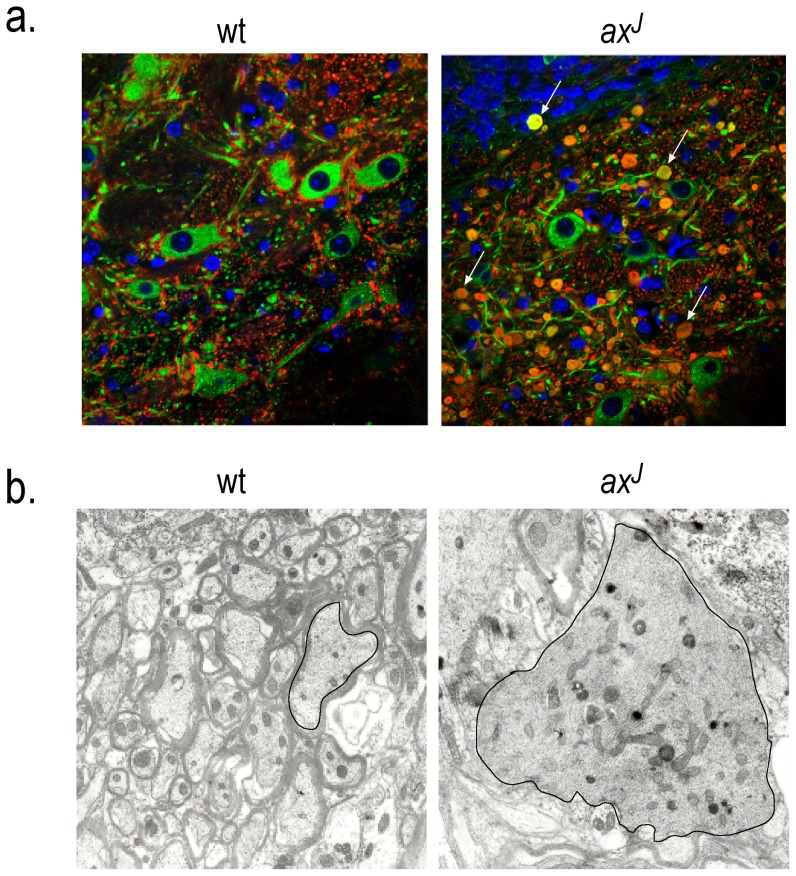
Structural analysis of the cerebellum from wt and *ax^J^* mice. (A) Indirect immunofluorescence of cerebellum from wt and *ax^J^* mice using calbindin (red), β-tubulin (green) and DAPI (blue). (B) Electron micrographs of cerebellar sections from wt and *ax^J^* mice. Black line indicates the outline of a Purkinje cell axon. Magnification at 2,500X. n = 3 to 4 animals per genotype.

Our previous studies demonstrated the presence of proximal Purkinje cell axonal swellings in the *ax^J^* mice [12]. To further examine the distribution of the axonal swellings in the *ax^J^* mice, we examined the distal Purkinje cell axons for any sign of disease. Consistent with our previous findings [12], we observed widespread Purkinje cell axonal swellings near the deep cerebellar nuclei in the *ax^J^* mice (Fig. 2a). Since increased tau phosphorylation correlates with decreased microtubule binding and disorganized microtubules [29], we used electron microscopy to compare the Purkinje cell axonal ultrastructure in the wt and *ax^J^* mice. The Purkinje cell axons from wt mice were myelinated and showed no gross structural alterations (Fig. 2b). Consistent with our immunohistochemical findings, large axonal swellings were observed along the Purkinje cell axons of the *ax^J^* mice. These swollen axons appeared to be unmyelinated and contained numerous mitochondria and randomly arranged microtubules (Fig. 2b). These changes in axonal structure did not correlate with any significant changes in the levels of α- or β-tubulin (Fig. 1a). Therefore, while there were no major changes in the steady-state levels of these microtubule proteins or tau, loss of Usp14 did result in aberrant localization of phosphorylated tau and microtubule disorganization in the *ax^J^* mice.

### *ax^J^* mice have elevated levels of phosphorylated tau in the cortex and hippocampus

**Figure 3:**
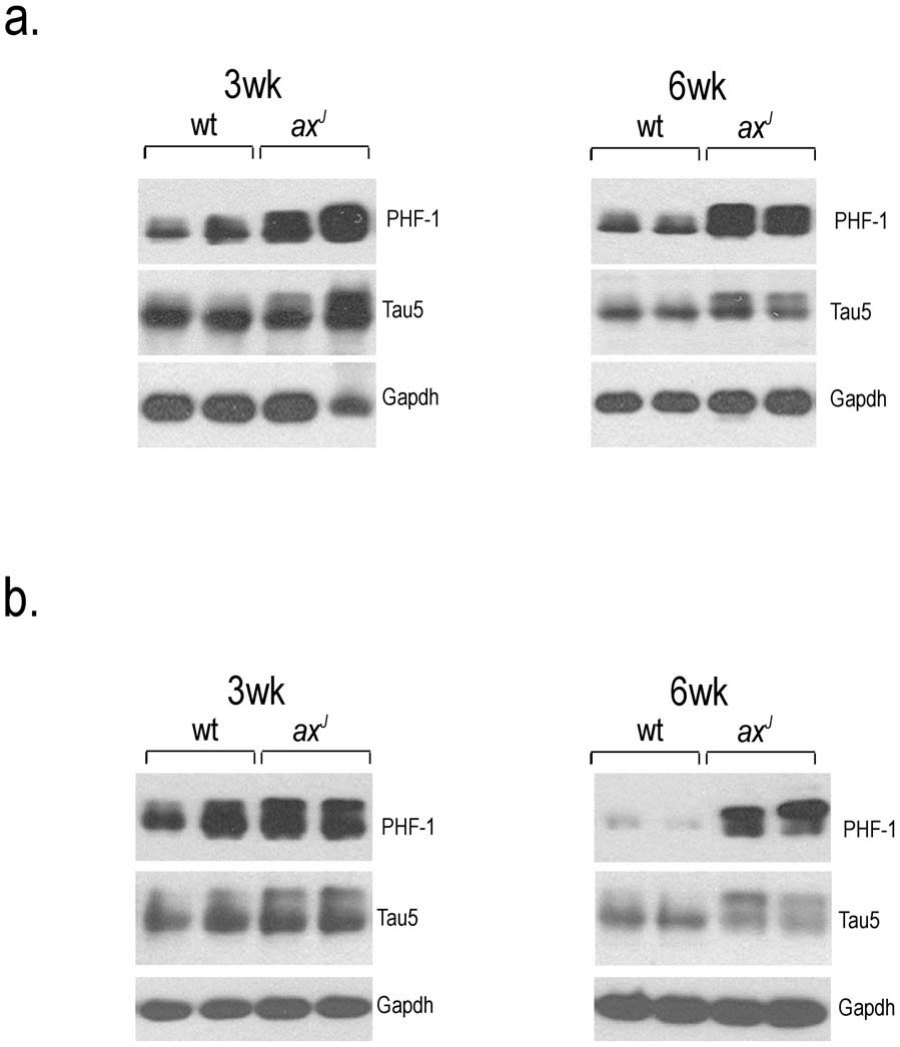
Immunoblot analysis of tau levels in specific brain regions. (A) Cortical and (B) hippocampal extracts were prepared from two different 3 and 6-week-old wt and *ax^J^* mice. The PHF-1 antibody was used to determine levels of phosphorylated tau, the tau5 antibody was used to detect total tau, and Gapdh was used as a loading control. n = 4 to 5 animals per genotype.

NFTs are found in several regions of the brains of AD patients, including the hippocampus and entorhinal and frontal cortexes [30]. To determine if frontal cortex and hippocampal brain regions of the *ax^J^* mice have increased levels of phosphorylated tau, we investigated PHF-1-reactive tau levels by immunoblot analysis. At both 3 and 6 weeks of age, elevated levels of PHF-1-reactive tau were observed in cortical and hippocampal extracts from the *ax^J^* mice compared to controls (Fig. 3), demonstrating that loss of Usp14 results in widespread changes in tau phosphorylation in the *ax^J^* mice.

### Examination of phospho-tau epitopes in *ax^J^* mice

**Figure 4:**
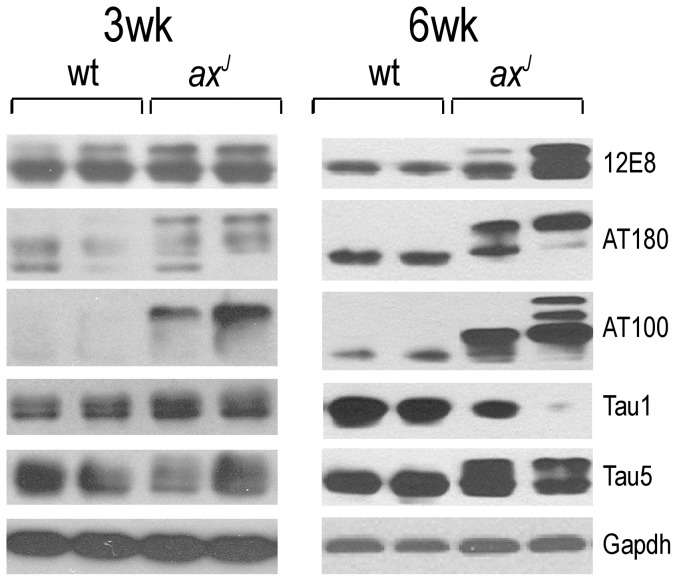
Analysis of phosphorylated tau-epitopes in wt and *ax^J^* mice. Hippocampal extracts from two different 3 and 6-week-old mice were probed with either the 12E8 antibody (pSer262), AT180 antibody (pThr231), AT100 antibody (pSer212/pThr214), tau1 antibody (unphosphorylated tau), or tau5 antibody (total tau). Gapdh was used as a loading control. n = 3 to 4 mice per genotype.

Several studies have investigated the phosphorylation state of tau as it relates to tau pathology, and a panel of antibodies has been generated to detect these different phosphorylation events on tau [26], [29]. Using these antibodies, we examined the levels of phospho-tau epitopes in hippocampal extracts from 3 and 6-week-old wt and *ax^J^* mice (Fig. 4). At 3 weeks of age, a significant increase in AT100 (pSer212 and pThr214) reactivity was observed in the *ax^J^* mice as compared to controls. By 6 weeks of age, there was a significant increase in reactivity with the AT100, AT180 (pThr231), and 12E8 (pSer262 and pSer356) phospho-tau epitopes and a significant decrease in reactivity with the non-phosphorylated tau1 epitope in the *ax^J^* mice as compared to controls. These results demonstrate that there are widespread changes in the phosphorylation state of tau in the Usp14-deficient *ax^J^* mice, and the time frame of these changes suggests that tau hyperphosphorylation may contribute to the axonal pathology found in the *ax^J^* mice.

### Dysregulation of GSK3β and stress kinases in *ax^J^* mice

**Figure 5:**
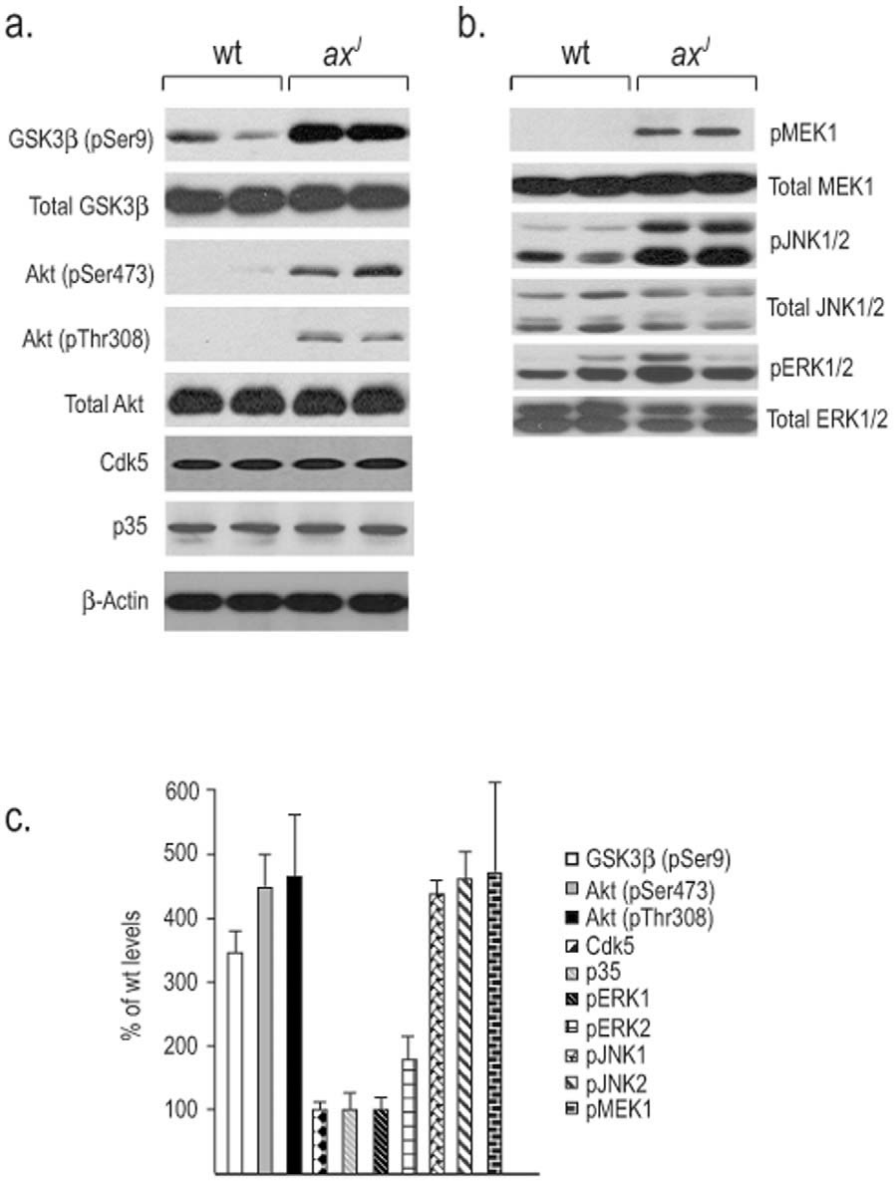
Immunoblot analysis of putative tau kinases in *ax^J^* and wt mice. (A) Steady-state levels of total and inactive (pSer9) GSK3β, total and activated (pSer473 and pThr308) Akt, Cdk5, p35 and β-actin in the hippocampus of two different wt and *ax^J^* mice. (B) Total and phosphorylated MAPKs MEK1, JNK1/2 and ERK1/2 were examined in hippocampal extracts from 4-week-old wt and *ax^J^* mice. (C) Graph depicts the quantitation of proteins from blots in A and B. n = 3 to 4 mice per genotype.

Several kinases, including Cdk5, GSK3β, ERK and JNK, have been implicated in the hyperphosphorylation of tau [23], [31]–[34], and these increases in tau phosphorylation are thought to accelerate NFT formation [35], [36]. To determine if the levels of these kinases are altered in the *ax^J^* mice, we examined hippocampal protein extracts from 6-week-old wt and *ax^J^* mice by immunoblotanalysis Fig. 5a). No changes in the levels of total GSK3β, Cdk5, or the Cdk5 activator p35 were seen in the extracts from the *ax^J^* mice as compared to controls. In contrast, a significant increase in the levels of the inactive form of GSK3β (pSer9) were observed in the *ax^J^* mice as compared to controls. Since the phosphorylation of GSK3β serine 9 is thought to be the result of activated Akt, we also investigated the levels of phosphorylated Akt (pSer473 and pThr308) in *ax^J^* and control mice. Consistent with the increased levels of pSer9 GSK3β, we observed an increase in both pThr308 and pSer473 Akt in the *ax^J^* mice. These results indicate that Cdk5 and GSK3β are not likely to be contributing to the increased levels of tau phosphorylation observed in the *ax^J^* mice.

Stress-activated kinases, which are activated by phosphorylation, have also been hypothesized to contribute to elevated phospho-tau levels in the brains of AD patients [23], [34], [37]–[40]. We therefore examined if loss of Usp14 alters the levels of stress-activated kinases in the *ax^J^* hippocampus (Fig. 5b). While no significant changes were observed in the levels of total MEK1, JNK or ERK, we did find increased levels of phosho-MEK1, JKN and ERK in the *ax^J^* mice (Fig. 5b). These results indicate that altered proteasome function caused by the loss of Usp14 results in widespread changes in the levels of activated stress kinases that have been implicated in tau phosphorylation.

### Tau reduction does not alter disease progression in the *ax^J^* mice

**Figure 6:**
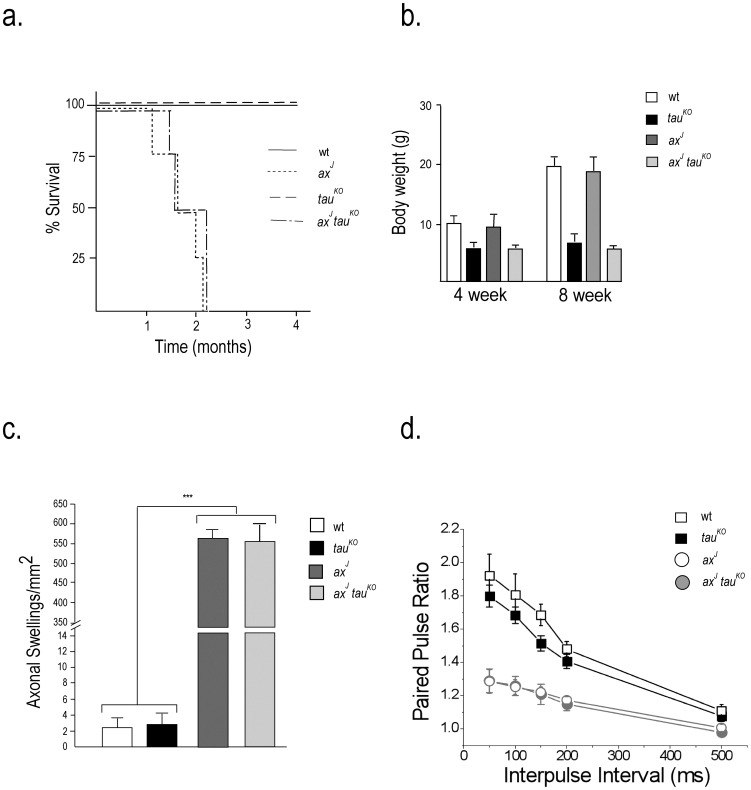
Effect of tau reduction on development and survival of *ax^J^* mice. (A) Four-month survival curves of wt, *ax^J^*, *tau^KO^* and *ax^J^tau^KO^* mice. *n* = 4 mice per genotype. (B) Body weights of 4 and 8-week-old wt, *ax^J^*, *tau^KO^* and *ax^J^tau^KO^* mice. *n* = 4 mice per genotype. (C) Purkinje cell axonal swellings were quantitated in wt, *ax^J^*, *tau^KO^* and *ax^J^tau^KO^* mice. n = 4 mice per genotype. ***indicates p≤10^−7^ (D) Paired-pulse facilitation was measured at hippocampal CA3-CA1 synapses. Facilitation was measured at 5 interpulse intervals ranging from 50 to 500 ms. n = 4 mice per genotype.

Hyperphosphorylated tau has been associated with several neurological disorders, and this aberrant phosphorylation of tau is believed to contribute to neuronal dysfunction [27], [41], [42]. Gene disruption studies indicate that removal of tau increases viability and improves synaptic function in some mouse models of AD [42], [43]. Since the *ax^J^* mice suffer from impaired neuromuscular development and synaptic dysfunction, that reduces their lifespan to approximately 2 months, we investigated if tau reduction would alter the expression of these phenotypes in the *ax^J^* mice. Examination of survival curves generated for wt and *tau^KO^* mice (Fig. 6a) demonstrated that genetic ablation of tau did not affect viability. When we examined the effect of removing tau from the *ax^J^* mice, we found that depletion of tau did not improve body weights or increase viability of the *ax^J^tau^KO^* mice as compared to *ax^J^* controls (Fig. 6a and b).

Our finding of PHF-1-reactive tau in the Purkinje cell processes of the *ax^J^* mice prompted us to investigate if tau depletion affected the number of Purkinje cell axonal swellings in the *ax^J^* mice. As previously reported [12], the *ax^J^* mice had a large increase in the number of Purkinje cell axonal swellings compared to wt controls (Fig. 6c), and there were no significant changes in the number of Purkinje cell axonal swellings in the *tau^KO^* mice as compared to controls (Fig. 6c). When we compared the number of axonal swellings in the *ax^J^* and *ax^J^tau^KO^* mice, we did not observe any significant changes in the abundance of these swellings, indicating that the increased levels of phospho-tau did not contribute to the formation of Purkinje cell axonal swellings in the *ax^J^* mice.

In addition to the structural deficits observed in the CNS of the *ax^J^* mice, loss of Usp14 also results in impairment of hippocampal short-term plasticity [13], [44] that is thought to be required for normal learning and memory. When we compared short-term plasticity in the hippocampus of wt, *tau^KO^*, and *ax^J^* mice, we only observed a reduction in paired-pulse facilitation in the *ax^J^* mice (Fig. 6d). No differences in paired-pulse facilitation were observed between the *ax^J^* and *ax^J^tau^KO^* mice (Fig. 6d), indicating that the synaptic changes caused by Usp14 deficiency are not due to alterations in tau phosphorylation.

## Discussion

The UPS regulates numerous cellular pathways by controlling protein abundance. This task is accomplished by the coordinated ubiquitination of proteins, followed by their destruction by the proteasome. Proteasomal deubiquitinating enzymes alter protein degradation rates by removing the ubiquitin side chains attached to substrates, thus preventing their degradation [14], [15], [45]. Usp14 is one of three deubiquitinating enzymes found on mammalian proteasomes, and blocking Usp14's deubiquitinating activity has been shown to accelerate degradation of aggregate-prone proteins *in vitro* [11], [14], [15]. However, our investigations on the loss of Usp14 *in vivo* did not reveal any significant changes in the steady-state levels of tau or ataxin-3 in the brains of the Usp14-deficient *ax^J^* mice compared to controls, indicating that endogenous tau and ataxin-3 may not be substrates for Usp14, or these proteins may be degraded through an alternative pathway *in vivo* [46]. In addition, the lack of any detectable change in these proteins may be due to decreased levels of free ubiquitin in the *ax^J^* mice that could reduce targeting of tau and ataxin-3 to the proteasome. Further experimentation will be required to determine if Usp14 plays a direct role in the degradation of specific classes of proteasomal substrates, such as proteins produced with mutated amino acid sequences.

Proteasome dysfunction induced by the loss of Usp14 results in a significant increase in the levels of phosphorylated tau in the brains of the *ax^J^* mice. We found increased levels of PHF-1-reactive tau that coincided with the presence of disorganized microtubules and Purkinje cell axonal swellings. These changes in tau were found in several regions of the *ax^J^* brain, suggesting that the loss of Usp14 results in a global change in kinase and/or phosphatase activity. The large increase in different reactive tau phospho-epitopes seen in the *ax^J^* mice is also consistent with this idea. The phospho-tau epitopes that were increased in the *ax^J^* mice include Thr212, Ser214, Thr231, Ser235, Ser262, and Ser356, and these phospho-epitopes are also detected in neurodegenerative diseases such as AD [26], [47]–[49]. Tau has been shown to associate with microtubules through internal repeat microtubule-binding domains, and these regions of tau are sites for protein phosphorylation [29]. Since phosphorylated tau is less effective than non-phosphorylated tau in binding polymerized microtubules, it is believed that the phophorylation state of tau can directly affect microtubule assembly and/or stability [50]. While tau is normally restricted to axons, PHF-1-reactive tau was also detected in the soma and dendrites of the Purkinje cells in the *ax^J^* mice. In addition, we also detected PHF-1-reactive tau in discrete focal regions in the *ax^J^* Purkinje cell axons. These changes in the phosphorylation and distribution of tau are hallmarks of AD, indicating that altered proteasomal function could be a contributing factor in the development of tau pathologies.

Aberrant phosphorylation of tau has been suggested to contribute to the accumulation of tau in neurons [51], and numerous protein kinases have been shown to interact with and phosphorylate tau [52]. For example, GSK3β can phosphorylate multiple cytoskeletal proteins including tau, MAP1b and APC [53]. Since GSK3β phosphorylation of tau and Map1b reduces their ability to bind microtubules, we reasoned that increased GSK3β kinase activity may contribute to the altered microtubule structure observed in the axons of *ax^J^* mice. Surprisingly, instead of observing increased GSK3β activity, we detected a 5-fold increase in the levels of inactive GSK3β, indicating that GSK3β is not likely to be responsible for the elevated levels of phosphorylated tau in the *ax^J^* mice. In addition, our studies demonstrated that the steady-state levels of Cdk5 and its activator p35 do not change in the *ax^J^* mice compared to wt controls. Like Cdk5 and p35, the steady-state levels of total GSK3β are also unchanged in the *ax^J^* mice. Acting upstream of GSK3β, Akt and PTEN have been shown to regulate the phosphorylation state of GSK3β. Consistent with an increase in inactive GSK3β, we observed a 3-fold increase in the levels of phospho-Akt. These alterations could result in widespread cellular changes in the *ax^J^* mice, including effects on protein and glycogen synthesis, apoptosis, and intracellular-signaling pathways such as WNT.

The ERK, JNK and p38 MAPK pathways are activated in neurons of patients with AD, which suggests that the MAPK pathways are involved in the pathogenesis of AD [54]. Our data indicate that the ERK and JNK pathways are also activated in the *ax^J^* mice. Although there is no evidence that activated ERK or JNK directly phosphorylate tau *in vivo*, several studies have demonstrated that altered proteasome activity leads to the activation of MAPKs [20]. Therefore, while increased tau phosphorylation caused by the activation of these signaling pathways does not contribute to pathogenesis, these altered signaling pathways may affect synaptic function in the *ax^J^* mice through a tau-independent mechanism.

Hyperphosphorylation of tau has been reported in many animal models of neurological disease [31], [33], [55], [56], but the relationship between increased levels of hyperphosphorylated tau and disease is not clear. Tau reduction provides a protective effect in several models of neurodegeneration, supporting the notion that tau can be deleterious to neurons. For example, genetic ablation of tau in an animal model of AD prevented the behavioral deficits and neuronal excitotoxicity induced by beta amyloid [42], [43]. Analysis of *ax^J^* mice with a genetic ablation of tau demonstrated that the reduced life span, Purkinje cell axonal swellings, and altered synaptic plasticity were not due to changes in tau. Even in the context of neurological disease, the presence of phosphorylated tau may only represent a marker of neuronal stress, as opposed being to a major factor contributing to neurological disease.

## References

[1] Glickman MH, Ciechanover A (2002) The ubiquitin-proteasome proteolytic pathway: destruction for the sake of construction. Physiol Rev 82: 373–428. 11917093 10.1152/physrev.00027.2001

[2] Bingol B, Sheng M (2011) Deconstruction for reconstruction: the role of proteolysis in neural plasticity and disease. Neuron 69: 22–32. 21220096 10.1016/j.neuron.2010.11.006

[3] Burke RE (2004) Recent advances in research on Parkinson disease: synuclein and parkin. Neurologist 10: 75–81. 14998437 10.1097/01.nrl.0000117822.90759.83

[4] Greer PL, Hanayama R, Bloodgood BL, Mardinly AR, Lipton DM, et al (2010) The Angelman Syndrome protein Ube3A regulates synapse development by ubiquitinating arc. Cell 140: 704–716. 20211139 10.1016/j.cell.2010.01.026 PMC2843143

[5] Wilkinson KD, Urban MK, Haas AL (1980) Ubiquitin is the ATP-dependent proteolysis factor I of rabbit reticulocytes. J Biol Chem 255: 7529–7532. 6249803

[6] Dawson TM, Dawson VL (2003) Molecular pathways of neurodegeneration in Parkinson's disease. Science 302: 819–822. 14593166 10.1126/science.1087753

[7] McNaught KS, Olanow CW, Halliwell B, Isacson O, Jenner P (2001) Failure of the ubiquitin-proteasome system in Parkinson's disease. Nat Rev Neurosci 2: 589–594. 11484002 10.1038/35086067

[8] Hanna J, Finley D (2007) A proteasome for all occasions. FEBS Lett 581: 2854–2861. 17418826 10.1016/j.febslet.2007.03.053 PMC1965587

[9] Finley D (2009) Recognition and processing of ubiquitin-protein conjugates by the proteasome. Annu Rev Biochem 78: 477–513. 19489727 10.1146/annurev.biochem.78.081507.101607 PMC3431160

[10] Lee MJ, Lee BH, Hanna J, King RW, Finley D (2011) Trimming of ubiquitin chains by proteasome-associated deubiquitinating enzymes. Mol Cell Proteomics 10: R110 003871. 10.1074/mcp.R110.003871 PMC3098602 20823120

[11] Guterman A, Glickman MH (2004) Deubiquitinating enzymes are IN/(trinsic to proteasome function). Curr Protein Pept Sci 5: 201–211. 15188770 10.2174/1389203043379756

[12] Crimmins S, Jin Y, Wheeler C, Huffman AK, Chapman C, et al (2006) Transgenic rescue of ataxia mice with neuronal-specific expression of ubiquitin-specific protease 14. J Neurosci 26: 11423–11431. 17079671 10.1523/JNEUROSCI.3600-06.2006 PMC6674543

[13] Wilson SM, Bhattacharyya B, Rachel RA, Coppola V, Tessarollo L, et al (2002) Synaptic defects in ataxia mice result from a mutation in Usp14, encoding a ubiquitin-specific protease. Nat Genet 32: 420–425. 12368914 10.1038/ng1006

[14] Lee BH, Lee MJ, Park S, Oh DC, Elsasser S, et al (2010) Enhancement of proteasome activity by a small-molecule inhibitor of USP14. Nature 467: 179–184. 20829789 10.1038/nature09299 PMC2939003

[15] Hanna J, Hathaway NA, Tone Y, Crosas B, Elsasser S, et al (2006) Deubiquitinating enzyme Ubp6 functions noncatalytically to delay proteasomal degradation. Cell 127: 99–111. 17018280 10.1016/j.cell.2006.07.038

[16] Mayer RJ, Lowe J, Lennox G, Doherty F, Landon M (1989) Intermediate filaments and ubiquitin: a new thread in the understanding of chronic neurodegenerative diseases. Prog Clin Biol Res 317: 809–818. 2557642

[17] Kosik KS, Joachim CL, Selkoe DJ (1986) Microtubule-associated protein tau (tau) is a major antigenic component of paired helical filaments in Alzheimer disease. Proc Natl Acad Sci U S A 83: 4044–4048. 2424016 10.1073/pnas.83.11.4044 PMC323662

[18] Paul S (2008) Dysfunction of the ubiquitin-proteasome system in multiple disease conditions: therapeutic approaches. Bioessays 30: 1172–1184. 18937370 10.1002/bies.20852

[19] Shi YY, Small GW, Orlowski RZ (2006) Proteasome inhibitors induce a p38 mitogen-activated protein kinase (MAPK)-dependent anti-apoptotic program involving MAPK phosphatase-1 and Akt in models of breast cancer. Breast Cancer Res Treat 100: 33–47. 16807678 10.1007/s10549-006-9232-x

[20] Zhang L, Ebenezer PJ, Dasuri K, Bruce-Keller AJ, Liu Y, et al (2009) Proteasome inhibition modulates kinase activation in neural cells: relevance to ubiquitination, ribosomes, and survival. J Neurosci Res 87: 3231–3238. 19565657 10.1002/jnr.22147 PMC2875064

[21] Um JW, Im E, Park J, Oh Y, Min B, et al (2010) ASK1 negatively regulates the 26 S proteasome. J Biol Chem 285: 36434–36446. 20843792 10.1074/jbc.M110.133777 PMC2978573

[22] Jantas D, Lorenc-Koci E, Kubera M, Lason W (2011) Neuroprotective effects of MAPK/ERK1/2 and calpain inhibitors on lactacystin-induced cell damage in primary cortical neurons. Neurotoxicology 32: 845–856. 21683092 10.1016/j.neuro.2011.05.013

[23] Zhu X, Lee HG, Raina AK, Perry G, Smith MA (2002) The role of mitogen-activated protein kinase pathways in Alzheimer's disease. Neurosignals 11: 270–281. 12566928 10.1159/000067426

[24] Dawson HN, Ferreira A, Eyster MV, Ghoshal N, Binder LI, et al (2001) Inhibition of neuronal maturation in primary hippocampal neurons from tau deficient mice. J Cell Sci 114: 1179–1187. 11228161 10.1242/jcs.114.6.1179

[25] Speed HE, Dobrunz LE (2009) Developmental Changes in Short-Term Facilitation Are Opposite at Temporoammonic Synapses Compared to Schaffer Collateral Synapses onto CA1 Pyramidal Cells. Hippocampus 19: 187–204. 18777561 10.1002/hipo.20496 PMC2631616

[26] Otvos L Jr, Feiner L, Lang E, Szendrei GI, Goedert M, et al (1994) Monoclonal antibody PHF-1 recognizes tau protein phosphorylated at serine residues 396 and 404. J Neurosci Res 39: 669–673. 7534834 10.1002/jnr.490390607

[27] Grundke-Iqbal I, Iqbal K, Quinlan M, Tung YC, Zaidi MS, et al (1986) Microtubule-associated protein tau. A component of Alzheimer paired helical filaments. J Biol Chem 261: 6084–6089. 3084478

[28] Gotz J, Ittner LM, Kins S (2006) Do axonal defects in tau and amyloid precursor protein transgenic animals model axonopathy in Alzheimer's disease? Journal of Neurochemistry 98: 993–1006. 16787410 10.1111/j.1471-4159.2006.03955.x

[29] Biernat J, Gustke N, Drewes G, Mandelkow EM, Mandelkow E (1993) Phosphorylation of Ser262 strongly reduces binding of tau to microtubules: distinction between PHF-like immunoreactivity and microtubule binding. Neuron 11: 153–163. 8393323 10.1016/0896-6273(93)90279-z

[30] Zubenko GS, Moossy J, Martinez AJ, Rao GR, Kopp U, et al (1989) A brain regional analysis of morphologic and cholinergic abnormalities in Alzheimer's disease. Arch Neurol 46: 634–638. 2730375 10.1001/archneur.1989.00520420054022

[31] Ahlijanian MK, Barrezueta NX, Williams RD, Jakowski A, Kowsz KP, et al (2000) Hyperphosphorylated tau and neurofilament and cytoskeletal disruptions in mice overexpressing human p25, an activator of cdk5. Proc Natl Acad Sci U S A 97: 2910–2915. 10706614 10.1073/pnas.040577797 PMC16029

[32] Wagner U, Utton M, Gallo JM, Miller CC (1996) Cellular phosphorylation of tau by GSK-3 beta influences tau binding to microtubules and microtubule organisation. J Cell Sci 109 (Pt 6) 1537–1543. 8799840 10.1242/jcs.109.6.1537

[33] Lucas JJ, Hernandez F, Gomez-Ramos P, Moran MA, Hen R, et al (2001) Decreased nuclear beta-catenin, tau hyperphosphorylation and neurodegeneration in GSK-3beta conditional transgenic mice. Embo J 20: 27–39. 11226152 10.1093/emboj/20.1.27 PMC140191

[34] Tatebayashi Y, Planel E, Chui DH, Sato S, Miyasaka T, et al (2006) c-jun N-terminal kinase hyperphosphorylates R406W tau at the PHF-1 site during mitosis. FASEB J 20: 762–764. 16478768 10.1096/fj.05-4362fje

[35] Jackson GR, Wiedau-Pazos M, Sang TK, Wagle N, Brown CA, et al (2002) Human wild-type tau interacts with wingless pathway components and produces neurofibrillary pathology in Drosophila. Neuron 34: 509–519. 12062036 10.1016/s0896-6273(02)00706-7

[36] Noble W, Olm V, Takata K, Casey E, O M, et al (2003) Cdk5 is a key factor in tau aggregation and tangle formation in vivo. Neuron 38: 555–565. 12765608 10.1016/s0896-6273(03)00259-9

[37] Ploia C, Antoniou X, Sclip A, Grande V, Cardinetti D, et al (2011) JNK plays a key role in tau hyperphosphorylation in Alzheimer's disease models. J Alzheimers Dis 26: 315–329. 21628793 10.3233/JAD-2011-110320

[38] Yoon SY, Park JS, Choi JE, Choi JM, Lee WJ, et al (2010) Rosiglitazone reduces tau phosphorylation via JNK inhibition in the hippocampus of rats with type 2 diabetes and tau transfected SH-SY5Y cells. Neurobiol Dis 40: 449–455. 20655383 10.1016/j.nbd.2010.07.005

[39] Harris FM, Brecht WJ, Xu Q, Mahley RW, Huang Y (2004) Increased tau phosphorylation in apolipoprotein E4 transgenic mice is associated with activation of extracellular signal-regulated kinase: modulation by zinc. J Biol Chem 279: 44795–44801. 15322121 10.1074/jbc.M408127200

[40] Pei JJ, Braak H, An WL, Winblad B, Cowburn RF, et al (2002) Up-regulation of mitogen-activated protein kinases ERK1/2 and MEK1/2 is associated with the progression of neurofibrillary degeneration in Alzheimer's disease. Brain Res Mol Brain Res 109: 45–55. 12531514 10.1016/s0169-328x(02)00488-6

[41] Morris M, Maeda S, Vossel K, Mucke L (2011) The Many Faces of Tau. Neuron 70: 410–426. 21555069 10.1016/j.neuron.2011.04.009 PMC3319390

[42] Roberson ED, Scearce-Levie K, Palop JJ, Yan F, Cheng IH, et al (2007) Reducing endogenous tau ameliorates amyloid beta-induced deficits in an Alzheimer's disease mouse model. Science 316: 750–754. 17478722 10.1126/science.1141736

[43] Roberson ED, Halabisky B, Yoo JW, Yao J, Chin J, et al (2011) Amyloid-beta/Fyn-induced synaptic, network, and cognitive impairments depend on tau levels in multiple mouse models of Alzheimer's disease. J Neurosci 31: 700–711. 21228179 10.1523/JNEUROSCI.4152-10.2011 PMC3325794

[44] Walters BJ, Campbell SL, Chen PC, Taylor AP, Schroeder DG, et al (2008) Differential effects of Usp14 and Uch-L1 on the ubiquitin proteasome system and synaptic activity. Mol Cell Neurosci 39: 539–548. 18771733 10.1016/j.mcn.2008.07.028 PMC2734958

[45] Lam YA, Xu W, DeMartino GN, Cohen RE (1997) Editing of ubiquitin conjugates by an isopeptidase in the 26S proteasome. Nature 385: 737–740. 9034192 10.1038/385737a0

[46] Berger Z, Ravikumar B, Menzies FM, Oroz LG, Underwood BR, et al (2006) Rapamycin alleviates toxicity of different aggregate-prone proteins. Hum Mol Genet 15: 433–442. 16368705 10.1093/hmg/ddi458

[47] Biernat J, Mandelkow EM, Schroter C, Lichtenberg-Kraag B, Steiner B, et al (1992) The switch of tau protein to an Alzheimer-like state includes the phosphorylation of two serine-proline motifs upstream of the microtubule binding region. Embo J 11: 1593–1597. 1563356 10.1002/j.1460-2075.1992.tb05204.x PMC556608

[48] Porzig R, Singer D, Hoffmann R (2007) Epitope mapping of mAbs AT8 and Tau5 directed against hyperphosphorylated regions of the human tau protein. Biochemical and Biophysical Research Communications 358: 644–649. 17499212 10.1016/j.bbrc.2007.04.187

[49] Goedert M, Jakes R, Crowther RA, Cohen P, Vanmechelen E, et al (1994) Epitope mapping of monoclonal antibodies to the paired helical filaments of Alzheimer's disease: identification of phosphorylation sites in tau protein. Biochem J 301 Pt 3: 871–877. 7519852 10.1042/bj3010871 PMC1137067

[50] Trinczek B, Biernat J, Baumann K, Mandelkow EM, Mandelkow E (1995) Domains of tau protein, differential phosphorylation, and dynamic instability of microtubules. Mol Biol Cell 6: 1887–1902. 8590813 10.1091/mbc.6.12.1887 PMC366657

[51] Grundke-Iqbal I, Iqbal K, Tung YC, Quinlan M, Wisniewski HM, et al (1986) Abnormal phosphorylation of the microtubule-associated protein tau (tau) in Alzheimer cytoskeletal pathology. Proc Natl Acad Sci U S A 83: 4913–4917. 3088567 10.1073/pnas.83.13.4913 PMC323854

[52] Dolan PJ, Johnson GV (2010) The role of tau kinases in Alzheimer's disease. Curr Opin Drug Discov Devel 13: 595–603. PMC2941661 20812151

[53] Scales TME, Lin S, Kraus M, Goold RG, Gordon-Weeks PR (2009) Nonprimed and DYRK1A-primed GSK3 beta-phosphorylation sites on MAP1B regulate microtubule dynamics in growing axons. J Cell Sci 122: 2424–2435. 19549690 10.1242/jcs.040162 PMC2704879

[54] Zhu XW, Castellani RJ, Takeda A, Nunomura A, Atwood CS, et al (2001) Differential activation of neuronal ERK, JNK/SAPK and p38 in Alzheimer disease: the ‘two hit’ hypothesis. Mechanisms of Ageing and Development 123: 39–46. 11640950 10.1016/s0047-6374(01)00342-6

[55] Asuni AA, Perry VH, O'Connor V (2010) Change in tau phosphorylation associated with neurodegeneration in the ME7 model of prion disease. Biochem Soc Trans 38: 545–551. 20298219 10.1042/BST0380545

[56] Treiber-Held S, Distl R, Meske V, Albert F, Ohm TG (2003) Spatial and temporal distribution of intracellular free cholesterol in brains of a Niemann-Pick type C mouse model showing hyperphosphorylated tau protein. Implications for Alzheimer's disease. J Pathol 200: 95–103. 12692847 10.1002/path.1345

